# Reproductive and obstetric outcomes in mosaic Turner’s Syndrome: a cross-sectional study and review of the literature

**DOI:** 10.1186/s12958-015-0055-7

**Published:** 2015-06-10

**Authors:** Emek Doğer, Yiğit Çakıroğlu, Yasin Ceylan, Esen Ulak, Özkan Özdamar, Eray Çalışkan

**Affiliations:** Department of Obstetrics & Gynecology, Kocaeli University School of Medicine, Kocaeli, Turkey; Department of Medical Genetics, Kocaeli University School of Medicine, Kocaeli, Turkey; Department of Obstetrics & Gynecology, Golcuk Military Hospital, Kocaeli, Turkey

**Keywords:** Mosaicisim, Turner’s syndrome, Obstetric outcome, Reproductive outcome

## Abstract

**Background:**

Turner’s syndrome (TS) is depicted as a total or partial absence of one X chromosome that results in ovarian dysgenesis. Chances of spontaneous pregnancy in TS are rare and the outcome of the pregnancies is known to be poor with an increased risk of miscarriage and stillbirths. Our aim is to evaluate reproductive and obstetric outcomes of natural conception and in-vitro fertilization (IVF) cycles in mosaic TS patients.

**Methods:**

A total of 22 mosaic TS cases (seventeen 45,X/46,XX and five 45,X/46,XX/47,XXX karyotypes) were evaluated.

**Results:**

Live birth and abortion rates were found as 32.7 % and 67.3 %, respectively in 52 pregnancies. Implantation, clinical pregnancy and take home baby rates were detected as 3.7 %, 8.6 % and 5.7 %, respectively per IVF cycle as a result of 35 cycles. Fecundability analysis revealed that 5 % of the cases experienced first pregnancy within 6 months and 8 % within the first 2 years. Mosaicism ratio did not have an effect on the time to the first pregnancy (*p* = .149).

**Conclusion:**

Only a small proportion of the mosaic TS patients conceive in the first 2 years of the marriage. Age of menarche and age of marriage appear not to have any impact on the chance of conceiving. Mosaic TS cases should counseled about the low odds of pregnancy and high miscarriage rates.

## Background

Turner’s syndrome (TS) is depicted as a total or partial absence of one X chromosome, and occurs in approximately 1/2200 of live born females [1]. Nearly 43-49 % of the patients are cases with classical TS who are monosomic for X chromosome (45,X). The remaining patients are mosaic cases carrying normal and abnormal cell lines together (most of them had 45,X/46,XX karyotype) (15-23 %), those with isochromosome in long arm of X chromosome (i(Xq)) (14 %), those with ring X chromosome (r(X)) (3-11 %) and those with 46,XX karyotype but having partial losses in one X chromosome (9 %). Y chromosome fragments are detected in 10-11 % of the cases [2–6].

Patients with classical TS demonstrate characteristic clinical features such as short stature, web neck, cardiovascular and renal abnormalities and hearing loss; and besides, gonadal dysgenesis in TS results in pubertal delay or failure, infertility and premature ovarian failure (POF) in most patients [7, 8]. Although correlation between genotype and phenotype is not well understood, mosaic cases present milder phenotypic abnormalities compared to those with 45,X karyotype [9]. Mosaic TS patients are more likely to experience normal pubertal development, regular menstrual cycles and to conceive spontaneously compared to those with 45,X monosomy [2]. Since mosaic patients are diagnosed following karyotype analysis due to recurrent pregnancy loss, repeated in vitro fertilization (IVF) failure and history of an abnormal offspring, our knowledge concerning reproductive and obstetric outcomes relies on case reports and case series [4, 10–12] and more comprehensive studies investigating the fertility outcomes of these patients.

The purpose of this study was to evaluate reproductive and obstetric outcomes of natural conception and IVF procedures in mosaic TS cases.

## Materials and methods

This retrospective study evaluated 706 female patients who underwent karyotyping between 2009–2013 in the laboratory of Medical Genetics Department of our tertiary health institution. The approval from the local ethics committee had been obtained prior to the initiation of the study. Informed consent from all individual participants included was obtained. Chromosomal analysis was performed by G-banding technique at high resolution. A hundred metaphases were counted for each patient and International System for Human Cytogenetic Nomenclature (ISCN, 2009) guidelines were used when performing karyotype analysis [13].

Mosaicism ratios of the cases were calculated by proportioning total number of abnormal cell lines. In karyotype analysis, mosaic cell line ratio of ≤ %10 was defined as low-grade mosaicism, and > %10 as high-grade mosaicism [14].

Medical history regarding prior hormone therapy, prior assisted reproductive techniques attempts in infertility cases, obstetric outcomes in patients who had conceived through spontaneous or IVF cycles and perinatal outcomes were obtained by face-to-face interview or assessment of hospital medical records. All patients were assessed with physical examination and underwent a set of diagnostic tests including thyroid function tests, abdominal ultrasonography and echocardiography.

### Statistical analysis

SPSS version 15.0 (Statistical Package for Social Science, Spss Inc. Chicago, IL, USA) was used for statistical analysis. Parametric variables were given as median (range), mean ± standard deviation or ± standard error. Chi-square test was used for the comparison of parametric variables. Non-parametric tests such as Mann–Whitney U test was used to compare non-parametric variables between cases with high-grade mosaicisim and low-grade mosaicism. Possibility of a total of 2-year fecundability was calculated at 6-month intervals by time-table analysis. The effect of menarche age, marriage age and mosaicism ratio on the time until spontaneous or IVF pregnancy was assessed by Cox-regression analysis. p < 0.05 was considered as statistically significant.

## Results

A total of 22 mosaic TS patients were extracted from 706 patients who underwent genetic karyotyping for varying indications including, recurrent implantation failure (5.1 %), recurrent pregnancy loss (defined as three or more consecutive miscarriage) (2.2 %), POF (2.2 %) and history of an offspring with any chromosomal or structural abnormality (4 %). Clinical characteristics of our study population were presented in Table 1. Menarche was achieved with hormone replacement therapy in three cases at the ages of 16,17 and 18 years and continued regularly. Menstruation was regular in 18 cases at the time of study enrollment whereas it was irregular in the two and the remaining two, who were diagnosed as POF at the ages of 28 and 38, were on hormone replacement therapy. There was one case with a short stature (<150 cm) phenotype and no cases with cardiovascular or renal abnormality, hearing loss and mental retardation among the included patients. The following systemic disorders were diagnosed in the patients; hypothyroidism in three, type 2 diabetes in three, asthma in one and generalized anxiety disorder in one patients. Uterine hypoplasia was observed in one case. In addition, one patient underwent hysteroscopic septum resection for uterine septum, another case underwent Strasmann metroplasty for bicornuat uterus and one underwent hysteroscopy and cavity expansion with fundal and lateral incisions for T-shaped uterus.

**Table 1:** Clinical characteristics of the study group

Patients age at the time of enrollment (years)^a^	37 (26–47)
Patients age at the time of diagnosis (years)^a^	34.5 (18–46)
Age of menarche (years)^a^	13 (11–18)
Age at marriage (years)^a^	25 (15–40)
Patients age at first pregnancy (years)^a,b^	23 (18–32)
Time from the marriage to the first conception (months)^a,b^	12 (6–49)
Height of the patients (cm)^a^	163 (132–174)
Body mass index at the enrollment (kg/m^2^)^c^	28.43 ± 1.21

^a^Data presented as median and range, ^b^In the cases who conceived spontaneously, ^c^ data presented as mean ± standart error

A total of 22 female patients, who were diagnosed as mosaic TS after karyotyping and who attended our IVF clinic with the diagnoses of recurrent implantation failure (*n* = 10), recurrent pregnancy loss (*n* = 9), infertility due to POF (*n* = 1), and history of a prior offspring with a chromosomal abnormality (*n* = 2), were included in the study. Out of 22 patients, five despite IVF treatment and one who never sought treatment, could not ever conceive. Out of remaining 16 cases, 11 conceived spontaneously and five conceived following IVF cycles; resulting in a total of 52 pregnancies of which 17 (32.7 %) resulted in live birth and 35 (67.3 %) resulted in abortion.

Of 22 mosaic TS patients’ karyotypes, 17 were 45,X/46,XX and five were 45,X/46,XX/47,XXX. There were no cases including 45,X/46,Xr(X); 45,X/46,X(i(Xq)); Y chromosome fragment or 46,XX karyotype with structural abnormalities in X chromosome. One patient was determined to have 45,X/46,XX inv(9)p11q13 karyotype. The comparison regarding the number and the percentages of pregnancies, miscarriages and live births between the different karyotypes of TS were presented in Table 2.

**Table 2:** Comparison of the ratios of live birth and miscarriage or terminated fetus between groups

Maternal karyotype	45,X/46,XX (*n* = 17)	45,X/46,XX/47,XXX (*n* = 5)	All cases (*n* = 22)	p
No of pregnancies (n)	35	17	52	.678
Live birth (n (%))	10 (28.6)	7 (41.2)	17 (32.7)	.611
Miscarriage (n (%))	25 (71.4)	10 (58.8)	35 (67.3)	.712

*p* < .05 Statistically significant

Mosaic cell line ratio was below 10 % in 17 and above 10 % in five cases. The comparison regarding the number and the percentages of pregnancies, miscarriages and live births between high-grade and low-grade mosaicism cases were presented in Table 3. Miscarriages included 29 spontaneous abortions, four biochemical abortions and two induced abortions (due to anencephaly and trisomy 21). Mean abortion week was found to be 8.16 ± 2.98 (±SD) in cases who experienced spontaneous abortion (Biochemical abortions were excluded from the calculation).

**Table 3:** Comparison of the ratios of live birth and miscarriage or terminated fetus between low and high grade mosaic cell line groups

Mosaic cell line ratio	Cases with low grade mosaic cell line (*n* = 17)	Cases with high grade mosaic cell line (*n* = 5)	All cases (*n* = 22)	p
No of Pregnancies (n)	46	6	52	.062
Miscarriage (n (%))	30 (65.2)	5 (83.3)	35 (67.3)	.468
Live Birth (n (%))	16 (34.8)	1 (16.7)	17 (32.7)	.127

*p* < .05 Statistically significant

A total of 82 embryo transfers were performed on 10 patients during 35 intracytoplasmic sperm injection (ICSI) treatment cycles. Five patients could not conceive despite ICSI and five cases could conceive following ICSI treatment. Out of these pregnancies, one clinical pregnancy resulted in spontaneous abortion, two pregnancies resulted in biochemical abortion and two live babies were taken home. Implantation rate per cycle was 3.7 % (3/82), clinical pregnancy rate was 8.6 % (3/35) and take home baby rate was 5.7 % (2/35). 90.4 % of the pregnancies (*n* = 47) (88.2 % of live born babies (*n* = 15)) have occurred in spontaneous cycles.

Perinatal outcomes of the 17 pregnancies that resulted in live birth are presented in Table 4. Mean birth weight of these newborns was found as 3355 ± 140 gr (±SE).

**Table 4:** Perinatal outcomes of the pregnancies that resulted in live birth

	Number	Percentage
Route of delivery		
Vaginal	9	52.9 %
Abdominal (C/S)	8	47.1 %
Fetal gender		
Female	12	70.6 %
Male	5	29.4 %
Adverse perinatal outcomes		
IUGR	1	5.9 %
P.previa	1	5.9 %
GDM	2	11.8 %

C/S, cesarean section; IUGR, intrauterine growth restriction; P.previa, placenta previa; GDM, gestational diabetes mellitus

Prenatal or postnatal cytogenetic examination was performed in three pregnancies, two as prenatal and one as postnatal investigations. Out of two prenatal cytogenetic examinations, one patient was diagnosed as Trisomy 21 and subsequently underwent a pregnancy termination; whereas the other revealed a normal karyotype. The postnatal investigation was of a patient who underwent karyotype analysis at the age of 15 due to mental retardation and a deletion was detected between the regions of 18q21.3 and q23. None of the miscarriages were evaluated genetically from the abortus materials.

Time table analysis revealed that pregnancy hazard rate within the first 2 years at 6-month intervals was found to be 0.01 in the first 6 months, 0.04 in the second 6 months, 0.02 in the third 6 months and 0.01 in the last 6 months. Neither spontaneous nor IVF pregnancy was detected beyond 60th month of marriage. COX regression analysis revealed that marriage age, menarche age and mosaicism ratio did not have an effect on the time until first pregnancy (*p* = .685; *p* = .350 and *p* = .149, respectively).

## Discussion

Gonadal dysgenesis in women with Turner Syndrome might depend on chromosome pairing failure during meiotic prophase, causing failure in synaptic formation at the zygotene and oocyte loss [15]. Majority of germ cells, which trigger spontaneous puberty in 10-30 % and provide pubertal development, start to diminish in the third month of intrauterine life, resulting in only 5-10 % of affected patients could menstruate regularly [16–19]. POF is another frequent clinical feature of TS and mean age of menopause was reported to be 29.3 years [18]. However; the degree of gonadal dysgenesis depends on the size of impaired regions of homologous chromosomes. Severe pairing failures induce the degeneration of all oocytes prior to puberty and are associated with rimary amenorrhea and poor sexual development, whereas mild pairing failures contribute to the survival of a considerable number of oocytes until puberty, leading to secondary amenorrhea and secondary impaired sexual development [15]. Thus, it is possible that puberty and reproductive capacities are less affected or even preserved in TS cases with mosaic karyotype [19]. When compared to the classical form, mosaic karyotype TS cases are more likely to present spontaneous puberty, normal levels of serum sex steroids and gonadotropins and follicles in ovarian biopsies [20]. In addition, the chance of spontaneous conceiving in women with TS was reported as 2-10 %, most of which are the cases of mosaic pattern and those with 45,X monosomy are candidates for oocyte donation [16, 21, 22].

X chromosome monosomy and mosaicism are encountered in 1.5 % of all amenorrhea cases although the incidence of X chromosome mosaicism in the general population still remains challenging [23]. In our study, we detected mosaic Turner karyotype in 2.2 % of the cases with a diagnosis of recurrent pregnancy loss and 5.1 % of the cases with recurrent implantation failure. Our study results are compatible with the previously published reports which indicated the rate of mosaic Turner karyotype in the recurrent pregnancy loss groups as 2.6 % [24]. X chromosome mosaicism ratio in female partner of ICSI candidate couples was reported to be 2.77-4.12 % whereas 45X/46XX mosaicism in couples with IVF failures due to severe male infertility and fertilization failures were reported to be 3.5-9.6 % [25–28]. Simpson et al. suggested that mosaicism has been underestimated as a cause of repeated failure in assisted reproduction [29].

The median age of marriage was 25 (15–40) and median age of first pregnancy in spontaneously pregnant women was 23 years (18–32) and these results are in agreement with the previous studies reporting median age of first pregnancy in TS syndrome cases as 23.5-27.2 years [4, 10]. ROC analysis revealed that marriage age had no impact on the chance of conceiving. Moreover, Cox-regression analysis demonstrated that the age of menarche, age of marriage and mosaicism ratio did not affect the time-to-first pregnancy. There were no significant difference between high-grade and low-grade mosaicism cases in terms of pregnancy, time-to-first spontaneous pregnancy, abortion and take home baby rates in our study. Several previously published reports indicated a correlation between mosaicism ratio and phenotypic abnormalities and reproductive capacity of the patients; on the contrary, some other publications did not find a consistent relationship [2, 30]. Scholtes et al. showed a correlation between mosaicism and a low implantation rate [31]. In contrast, Sonntag et al. could not find any significant impact of low-grade mosaicism on the course or outcome of ICSI in 20 couples [14]. No required minimum percentage of abnormal cells has been established to define true versus “low-grade” mosaicism. Thus, some studies disregard the importance of low-grade mosaicism [26]. In our study, first two-year fecundability analyses revealed that 5 % of the cases experienced their first pregnancy within 6 months and 8 % within first 2 years. No pregnancy was detected after 60 months of marriage. With the expectation of reduction in ovarian reserve with advanced age in TS, our results revealed that chance of fertility in cases with mosaic karyotype was high at younger ages but their chance will decrease if they do not conveive within the first 2 years.

Majority of TS cases who conceive spontaneously are known to have mosaic cell lines. In the study by Birkebaek et al., evaluating 410 Danish women with TS, 27 out of 31 women who could spontaneously conceive, at least once, had a TS diagnosis [4]. Similarly in the study by Bryman et al. there were 45,X/46,XX mosaicism in 25 out of 27 Swedish women with TS. While 23 cases had a spontaneous pregnancy, three cases conceived by assisted reproductive technologies [32]. Clinical pregnancy rate and implantation rate were reported to be 46 % and 30 %, respectively, by fresh embryo transfer in oocyte donation cycles, and 28 % and 19 %, respectively, in frozen embryo transfers in TS cases [33]. Pregnancy rates of these cases were found to be comparable with other women in donation programs or probably lower due to diminished endometrial receptivity [34, 35].

Many studies have shown that TS women who are able to conceive are at increased risk for miscarriage, stillbirths and malformed babies [7, 10, 36, 37]. Tarani et al. analyzed 160 spontaneous pregnancies in 74 women with TS and reported that 67.3 % of the pregnancies with 45,X/46,XX karyotype and 70.8 % of the pregnancies with 45,X/46,XX/47,XXX karyotype resulted in miscarriage or malformed fetus [10]. Similarly, Bryman et al. have reported that 45 % of cases with mostly 45,X/46,XX mosaicism who conceived with their own oocytes ended with miscarriage and 10 % of them with induced abortion [32]. Moreover, Kuo et al. have reported miscarriage rate as 68.6 % in patients with diminished ovarian reserve and 44.1 % in patients without diminished ovarian reserve among cases who had a history of prior recurrent spontaneous abortions with X chromosome mosaicism [38]. Among our patients, 67.3 % of the pregnancies were resulted in abortion or termination. However karyotyping was performed for nine cases in our study group due to recurrent pregnancy loss. This condition was a reason of how we detected a high abortion rate and besides, this rate is quite higher than the abortion rate of 10-15 % in the population [39].

Miscarriages that are frequently seen in TS cases are explained by chromosomal abnormalities in fetus, autoimmune disorders, ovarian and uterine factors [10, 11, 38, 40]. Aborted fetuses of TS women or their live born children are more susceptible to trisomy 21 (4 % vs 0.4 %, respectively) and TS (15 % vs 0.5 %, respectively) risks compared to general population [10]. Hereditary in nature was reported in mosaic TS cases and especially cases with ring chromosome [10, 41]. Singh et al. revealed in their study investigating 97 conceptions in 31 pregnant women with sex chromosome mosaicism that 75 % of the fetuses were abnormal and 50 % of these pregnancies were resulted in spontaneous abortion and 25 % had genetic or congenital abnormalities [42]. Besides, it was reported that abnormal karyotype ratio was increased from 42.9 % up to 73.7 % in abortion samples in the presence of diminished ovarian reserve [38]. Birkebaek et al. detected chromosomal aberrations in 6 of 25 pregnancies who underwent prenatal or postnatal cytogenetic analysis among TS cases with classical and mosaic forms [4]. In our study fetal karyotype analysis was not performed in all pregnancies terminated by abortion, hence the exact abnormality rates cannot be predicted however it seems likely to be higher. Cerebral paresis, neuropsychological disorder, aortic coarctation, cleft lip and palate and congenital tumor were detected in 5 (7 %) of 68 children born to women with TS [32]. Even mosaic, preimplantation diagnosis, chorionic villus sampling or amniocentesis should be recommended for the patients undergoing infertility treatment if pregnancy is planned with their own oocytes since biological children of TS women are under risk for chromosomal abnormalities; and their children should be investigated for birth defects after delivery.

Uterine hypoplasia and related reduced uterine perfusion secondary to significant changes in utero-ovarian vascular anatomy, and subclinical uterine abnormalities in TS cases have been implicated in the etiology of miscarriages [33, 38, 43]. Although abortion rate is higher in pregnant women conceived with their own oocytes, use of donor oocytes does not reduce pregnancy loss rate in mosaic TS cases who underwent IVF (45 % with their own oocytes vs 26-30 % with donor oocytes) [32, 44]. This condition can be suggested as the evidence of the effect of diminished endometrial receptivity as well as oocyte-associated factors in TS cases. In our study group, uterus hypoplasia was present in one case and surgery-corrected uterine abnormality was present in three cases. It has been reported that uterine size were often normal in cases with mosaic karyotype and that they experience spontaneous puberty [45, 46]. Khastgir et al. have reported bicornuat uterus in four (13.8 %) of 29 TS cases, 10 of whom had mosaic karyotypes and miscarriage rate associated with uterus hypoplasia, bicornuat uterus and low fertilization rate was 50 % [34]. Kuo et al. have reported that they detected uterine abnormality in 16.7 % of mosaic X-chromosome aneuploidies with a history of recurrent spontaneous abortions and 5.2 % in the control group [38]. In our study, we found the ratio of Mullerian abnormality in all mosaic TS cases as 13.6 % and all pregnancies had terminated with abortions before the surgical corrections of the malformations. However, after the surgical corrections, four pregnancies were achieved and three terminated with delivery. These results suggest that the rate of uterine abnormalities in TS cases is high and that they may benefit from the surgery.

TS patients may experience complications during pregnancy due to congenital malformations and endocrine diseases, and should be evaluated for the presence of these pathologies before IVF or pregnancy [7]. Gestational diabetes mellitus was present in two (11.8 %) of 17 pregnancies that achieved live birth. These rates are close to those reported by Bryman et al. who affirmed a pregnancy induced hypertension and gestational diabetes rate of 5 % in TS cases, similar to the rate in general population [32, 47, 48]. In our study population, there was one pregnancy with a history of SGA fetus delivery (5.9 %) and none of the cases had preterm delivery. In TS, preterm delivery rate was reported between 8–37.1 % and low birth weight was between 8.8-27.5 % [49, 50]. Given that the chromosomal aberrations arise possibly from the transmission of the imbalances in the genetic arrangements in mother to the offsprings (10), genetic counseling should be offered to all who conceived with autologous oocytes (11). Preimplantation genetic diagnosis (PGD) may improve the chance of conceiving in patients with recurrent ART failure and TS (12).

Limitations of our study include its retrospective design, presence of some data that are based on patients’ memory, absence of a control group and inability to perform a karyotype analysis in all newborns. In the context of lack of a control group, we should emphasize that it was not possible to constitute a control group since the present was not a population-based study. In addition, it is obvious that it is difficult to conduct such a study in this issue in a prospective fashion. Besides, maternal age significantly affects gains and losses in sex chromosomes [51–53]. A significant correlation was reported between maternal age and incidence of 45,X cell after 51 years old, and incidence of 45,X cell was reported to be 3.2-5.1 % among the women older than this age [52, 54]. Although cases in our study were younger than 46 years old, mosaicism ratio in some cases, whose pregnancy age was young but diagnosis age was advanced, may not reflect exact karyotype profile. Moreover, in the study by Hanson et al. percentage of cases with mosaic karyotype was found to be 45 % only with karyotyping but it reached to 70 % when FISH was used [3]. Mosaicism ratio was given by the assessment of 100 metaphase plaques in all cases in our study, and it should be considered that FISH method was not used while evaluating our results. Last, the results obtained in the present study might have been affected by the non-mosaicism-related individual factors given that some patients included had more than one pregnancy as we stressed that 16 cases experienced 52 pregnancies.

## Conclusion

Only a small proportion of the mosaic TS patients conceive in the first 2 years of marriage, hence any possible interventions should be considered within this period and at as much younger ages as possible. In TS patients who conceived, only 5.7 % take home baby whereas 67.3 % abort. Age of menarche and age of marriage appear not to have any impact on the chance of conceiving. Mosaicism ratio does not affect the time to the first pregnancy. Patients should be informed about high abortion rates after pregnancy.

## Abbreviations

TS: Turner’s Syndrome
IVF: In Vitro Fertilization
ICSI: Intra Cytoplasmic Sperm Injection
FISH: Fluorescent In Situ Hybridization
